# Utilization of lignocellulosic hydrolysates for photomixotrophic chemical production in *Synechococcus elongatus* PCC 7942

**DOI:** 10.1038/s42003-023-05394-w

**Published:** 2023-10-09

**Authors:** Jake N. Gonzales, Tanner R. Treece, Stephen P. Mayfield, Ryan Simkovsky, Shota Atsumi

**Affiliations:** 1 Plant Biology Graduate Group, University of California, Davis, Davis, CA 95616 USA; 2 Department of Chemistry, University of California, Davis, Davis, CA 95616 USA; 3 Division of Biological Sciences, University of California, San Diego, La Jolla, CA 92093 USA; 4 California Center for Algae Biotechnology, University of California, San Diego, La Jolla, CA 92093 USA

**Keywords:** Metabolic engineering, Bacterial synthetic biology

## Abstract

To meet the need for environmentally friendly commodity chemicals, feedstocks for biological chemical production must be diversified. Lignocellulosic biomass are an carbon source with the potential for effective use in a large scale and cost-effective production systems. Although the use of lignocellulosic biomass lysates for heterotrophic chemical production has been advancing, there are challenges to overcome. Here we aim to investigate the obligate photoautotroph cyanobacterium *Synechococcus elongatus* PCC 7942 as a chassis organism for lignocellulosic chemical production. When modified to import monosaccharides, this cyanobacterium is an excellent candidate for lysates-based chemical production as it grows well at high lysate concentrations and can fix CO_2_ to enhance carbon efficiency. This study is an important step forward in enabling the simultaneous use of two sugars as well as lignocellulosic lysate. Incremental genetic modifications enable catabolism of both sugars concurrently without experiencing carbon catabolite repression. Production of 2,3-butanediol is demonstrated to characterize chemical production from the sugars in lignocellulosic hydrolysates. The engineered strain achieves a titer of 13.5 g L^−1^ of 2,3-butanediol over 12 days under shake-flask conditions. This study can be used as a foundation for industrial scale production of commodity chemicals from a combination of sunlight, CO_2_, and lignocellulosic sugars.

## Abstract (web summary)

*Synechococcus elongatus* PCC 7942 was modified to import and use monosaccharides, which allows it to grow on lignocellulosic lysate and produce 2,3-butanediol.

## Introduction

The increase in climate volatility due to overuse of fossil fuels has kindled a multidisciplinary effort to develop sustainable, biologically derived fuels and other commodity chemicals often by leveraging synthetic biology and metabolic engineering of microbes for the fermentation of agricultural products most typically from fermentation of agricultural products^1^. However, the global food system is increasingly under pressure from a burgeoning world population, meaning the use of edible agricultural products must be limited to meeting food demands, while alternative feedstocks must be used for precision fermentation^2^. The United States generates 368 million metric tons of biogenic waste biomass every year with the potential to expand up to 1 billion metric tons^3^. Much of this is in the form of non-edible lignocellulosic crop waste, representing a promising, underutilized carbon pool^4^. Unlocking the practical use of this biomass has become a major research priority for government agencies such as the Department of Energy^3^. Glucose and xylose, the primary components of lignocellulose lysates, would be ideal substrates for commodity chemical production^5^. While the process of efficient valorization of monosaccharide sugars in corn stover and other crop wastes continues to improve, harsh pretreatments of lignocellulose is often required to release fermentable sugars and this process releases several inhibitors of microbial growth such as furan derivatives^6–9^. To increase tolerance to lignocellulose-derived feedstocks, other studies have utilized strategies such as adaptive laboratory evolution and introducing engineered oxireductases^10^. Additionally, despite the relative abundance of lysate feedstocks, traditional heterotrophic hosts struggle to efficiently co-utilize multiple sugars at once due to carbon catabolite repression (CCR), which requires substantial metabolic engineering to overcome^11–13^. The sensitivity of traditional production hosts to CCR and toxic lignocellulosic feedstocks prevents efficient industrial utilization. These limitations necessitate further innovation. This study explores a photomixotrophic production strategy in 7942 utilizing two sugars concurrently. This system was found to catabolize xylose and glucose with no apparent CCR, grow well in high lignocellulosic lysate concentrations, and utilize sugars from lignocellulosic lysate to make an industrially relevant product.

The cyanobacterium *Synechococcus elongatus* PCC 7942 (hereafter 7942) is an ideal candidate for lysate-based production systems. Photosynthetic microorganisms, such as cyanobacteria and algae, have lagged behind in development as production chassis due to the inherent productivity limitations of photosynthesis^14,15^. Increasing the efficiency of ribulose-1,5-bisphosphate carboxylase/oxygenase (RuBisCO), the rate-limiting step of carbon fixation, is extremely difficult due to the context in which it evolved^16^. These limitations may explain why photoautotrophic growth of 7942 is relatively slow, with a doubling time of 6-7 hours^17^. This slow growth rate is prohibitive for use in industrial applications. One way in which to bypass this problem is through photomixotrophic growth. Photomixotrophy, the ability to co-utilize photosynthetically fixed CO_2_ and environmentally available sugars, combines the rapid product synthesis rate characteristic of heterotrophs and the photoautotrophic ability to fix CO_2_ from the atmosphere. These complimentary metabolic strategies allow for flexibility depending on environmental condition and the needs of the user. If sugars are not available, production will continue at a lower rate from fixed CO_2_. If light is not available, sugars can be used to completely support growth and product synthesis. Despite the added versatility of photomixotrophy, high cell density may inhibit the light reactions of photosynthesis and the system may favor heterotrophic growth. This study seeks to further improve on the versatility of photomixotrophy by engineering mixed sugar consumption and characterize lignocellulosic lysate.

While wild type 7942 is an obligate photoautotroph, it has been previously engineered to utilize externally provided sugars, such as glucose and sucrose, for growth^18,19^, allowing 7942 to grow in the dark while ameliorating the inherent limitations of obligate heterotrophs/autotrophs for bioproduction. As proof of concept, photomixotrophic 2,3-butanediol (23BD) production has been achieved in 7942 (ref. ^20^). 23BD is an ideal target product because of its short and simple biosynthetic pathway from the central metabolite pyruvate, its relatively non-toxic and non-volatile properties, and its projected 300-million-dollar market by 2030 (ref. ^21^). Additional metabolic engineering of the Calvin Benson Bassham cycle (CBB) improved titer and production rate drastically, ameliorating the supposed inherent limitations of photosynthetic production hosts^22^. It was found that increasing carbon flux through the CBB by engineering sugar catabolism in cyanobacteria can increase CO_2_ fixation rate without requiring RuBisCO optimization^22^. Photomixotrophy utilizing glucose and xylose has also been established in the cyanobacterium *Synechocystis* sp. PCC 6803 (hereafter 6803), yet 6803 was found to have lower xylose consumption rate in the presence of glucose suggesting difficulty catabolizing two sugars at once^23^.

In this work, we sought to engineer 7942 to co-utilize the two primary components of lysate, glucose and xylose in addition to CO_2_ to produce 23BD. To this end, we introduced sugar importers to 7942 and explored deregulation of primary metabolism. As an obligate photoautotroph, 7942 was found to not require additional efforts to overcome regulation that could lead to CCR. This is because the common mechanisms responsible for CCR in other bacteria such as the transcription activator receptor protein, the phosphoenolpyruvate–carbohydrate phosphotransferase system (PTS), and catabolite control protein A are not found in the 7942 genome^24,25^. In all, 7942 was found to grow without inhibition in lysate conditions exceeding 50 g L^−1^ of mixed sugars, while *E. coli* was observed to suffer an extensive growth detriment in even low lysate conditions (~5 g L^−1^). In the context of mixed sugar co-import, metabolic engineering of critical components in the CBB improved chemical production without reducing biomass accumulation. Finally, product yields were further improved through culture optimization, leading to maximum titers of 13.5 g L^−1^ 23BD and a theoretical maximum yield of 42% from lignocellulose hydrolysate feedstocks.

## Results and discussion

### Establishing photomixotrophic co-utilization of glucose and xylose

**Fig. 1:**
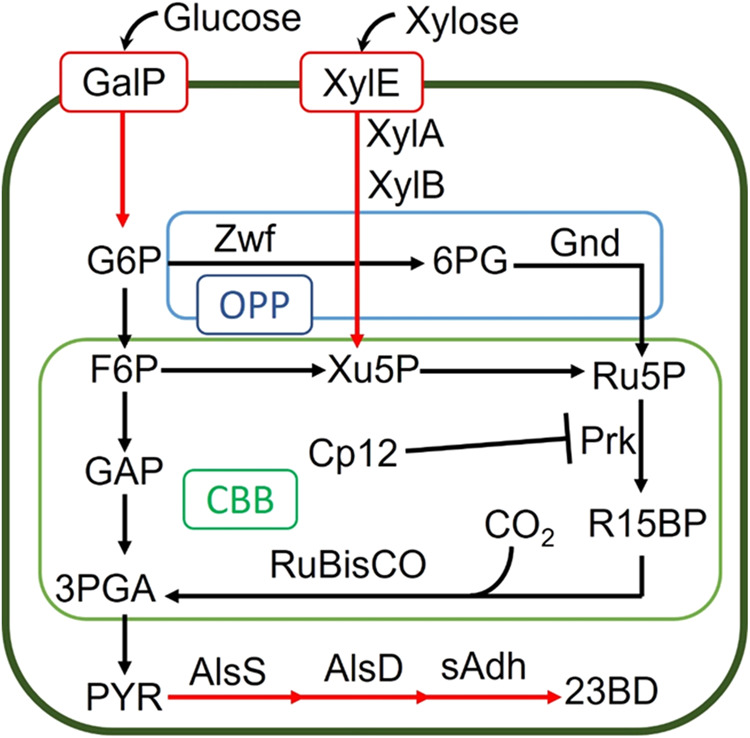
Photomixotrophic 23BD Pathway. An abbreviated schematic of glycolysis, the oxidative pentose phosphate pathway (OPP, blue) and the Calvin-Benson-Bassham cycle (CBB, green). Black lines are indicative of native enzymes. Red lines indicate heterologous genes expressed in the engineered strains. Metabolite abbreviations are as follows: G6P glucose-6-phosphate, F6P fructose-6-phosphate, 6PG 6-phosphoglycerate, Xu5P xylulose-5-phosphate, Ru5P ribulose-5-phosphate, R15BP ribulose-1,5 bisphosphate, GAP glyceraldehyde-3-phosphate, 3PGA 3-phosphoglycerate, PYR pyruvate, 23BD 2,3-butanediol. Enzyme abbreviations are as follows: GalP galactose-proton symporter, XylEAB xylose-proton symporter, xylose isomerase, xylulose kinase, Zwf glucose-1-phosphate 1-dehydrogenase, Gnd 6-phosphogluconate dehydrogenase, PRK phosphoribulokinase, RuBisCO ribulose bisphosphate carboxylase/oxygenase, AlsS acetolactate synthase, AlsD acetoin decarboxylase, Adh secondary alcohol dehydrogenase.

A strain capable of consuming the primary monosaccharides in cellulosic hydrolysates, glucose and xylose, was constructed from an existing xylose photomixotrophic 23BD production strain of 7942 (AL3417, Fig. 1 and Table 1)^18,20^. The 23BD production pathway consists of *alsS, alsD*, and *sadh*^26^. The *alsS* gene encodes an acetolactate synthase which converts two molecules of pyruvate into (*S*)-2-acetolactate^27^. 2-Acetolactate is decarboxylated to (*R*)-acetoin by an acetoin decarboxylase encoded by *alsD*^26^. Lastly, the *sadh* gene encoding a secondary alcohol dehydrogenase reduces (*R*)-acetoin to (*R,R*)−23BD^26^. The xylose catabolic pathway was derived from *E. coli*, and is composed of *xylE, xylA*, and *xylB*. The *xylE* gene encodes a proton symporter which facilitates pH-dependent uptake of xylose^28^. The *xylA* and *xylB* genes encode a xylose isomerase and kinase respectively; this pathway converts xylose into the intermediate of the pentose phosphate pathway, xylulose 5-phosphate^29^.

**Table 1:** Strains used in this study.

Strain	Genotype	Glucose uptake	Xylose uptake	23BD production	Source
*E. coli* MG1655	F- lambda- *ilvG*- *rfb*-50 *rph*-1	+	+	−	^37^
AL257	*Synechococcus elongatus* PCC 7942	−	−	−	^45^
AL3417	AL257 + NSI:: *lacI*^*q*^; *P*_trc_: *xylE-xylA-xylB*; Spec^R^, NSIII:: *lacI*^*q*^; *P*_trc_: *alsD-alsS-adh*; Gent^R^	−	+	+	^20^
AL3870	AL3417 + NSII:: *lacI*^*q*^; *P*_trc_: *galP-zwf-gnd*, Kan^R^	+	+	+	This study
AL4050	AL257 + NSI:: *lacI*^*q*^; *P*_trc_: *xylE-xylA-xylB*; Spec^R^, NSII:: *lacI*^*q*^; *P*_trc_: *galP-zwf-gnd*; Kan^R^	+	+	−	This study
AL4173	AL3417+ *cp12*:: *P*_*LlacO1*_*: prk P*_*trc*_: *galP-zwf-gnd*, Kan^R^	+	+	+	This study

The *galP* gene, encoding a glucose/galactose proton symporter from *E. coli*, was introduced to AL3417 (Fig. 1 and Table 1). Sugar import in *E coli* and many heterotrophs is primarily managed by the phosphoenolpyruvate-carbohydrate phosphotransferase system (PTS). Although this system is highly efficient, it is extremely sensitive to CCR and consumes phosphoenolpyruvate^30^. GalP, in contrast, transports glucose and/or galactose utilizing a proton gradient and is insensitive to the presence of other sugars making it ideal for mixed sugar systems. After transport across the cellular membrane, a native glucokinase then phosphorylates glucose, allowing integration into primary metabolism^31^. The *zwf* and *gnd* genes from *E. coli* encoding for an NADP^+^-dependent glucose-6-phosphate dehydrogenase and a 6-phosphogluconate dehydrogenase, respectively, were also introduced into AL3417, creating AL3870 (Fig. 1 and Table 1). The additional expression of *zwf* and *gnd* was previously demonstrated to improve CO_2_ fixation in the presence of glucose uptake by directing carbon flux toward the RuBisCO substrate, Ribulose 1,5-bisphosphate (R15BP)^22^.

**Fig. 2:**
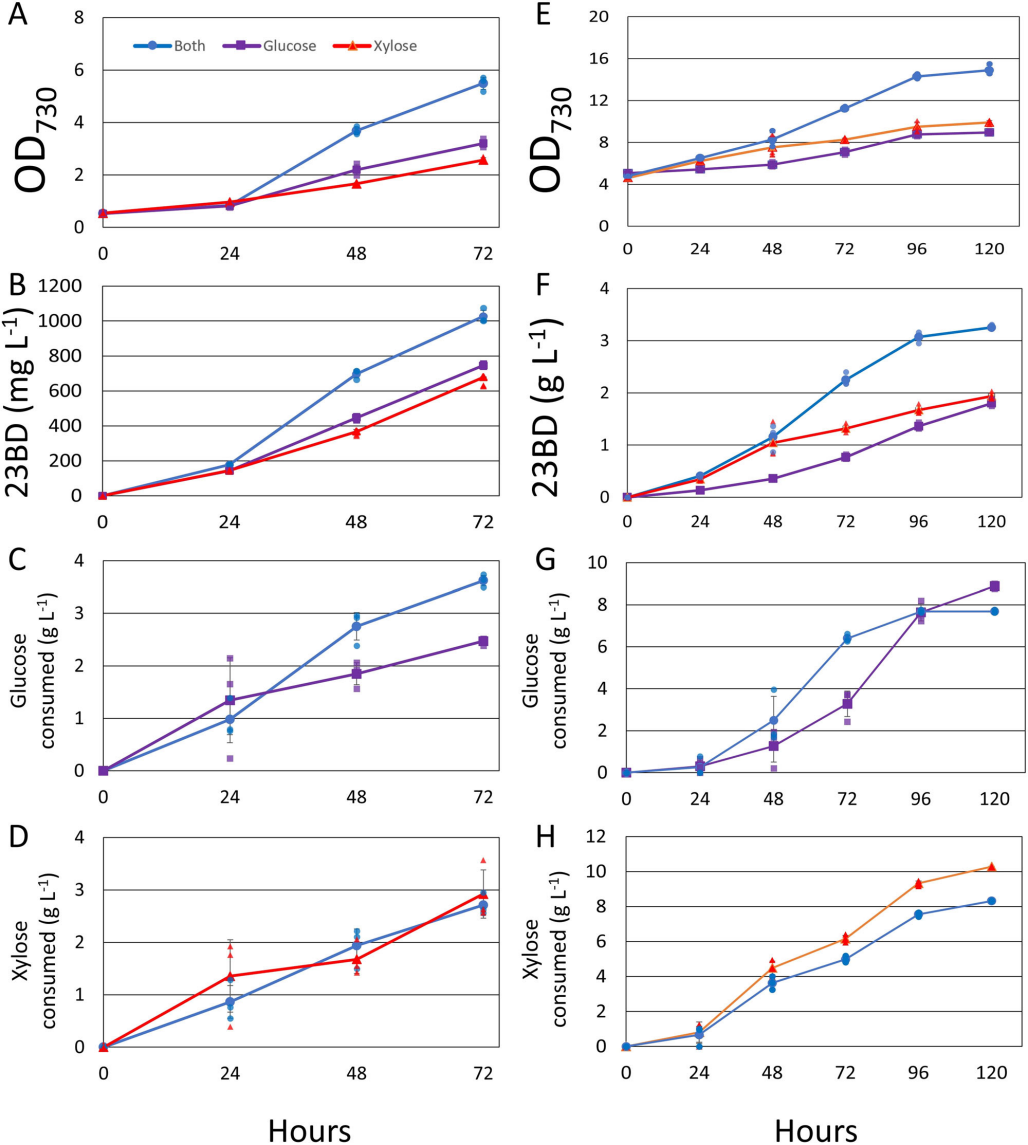
2,3-butanediol production from mixed sugar substrates. **A**–**D** Cells were cultured in BG-11 containing 5 g L^−1^ glucose (purple), xylose (red) or both (blue). Growth (**A**), 23BD production (**B**), glucose consumption (**C**), and xylose consumption (**D**) were monitored over 72 h. **E**–**H** Cells were grown in 5x BG-11 containing10 g L^−1^ glucose (purple), xylose (red), or both (blue). Growth (**E**), 23BD production (**F**), glucose consumption (**G**), and xylose consumption (**H**) were monitored over 120 h. *N* = 3 biological replicates; error bars represent standard deviation.

To investigate growth and 23BD production with glucose and/or xylose of AL3870, AL3870 was cultured in BG-11 media containing 5 g L^−1^ glucose, 5 g L^−1^ xylose, or 5 g L^−1^ of both under constant illumination (Fig. 2). Additionally, 20 mM NaHCO_3_ was added as a supplementary CO_2_ source. Growth and 23BD production were improved by the presence of both sugars compared to the single sugar conditions (Fig. 2A, B). A final titer of 1.0 g L^−1^ of 23BD was achieved over 72 h in the presence of both sugars (Fig. 2B). The single sugar conditions achieved 23BD titers of 0.75 and 0.68 g L^−1^ for glucose and xylose conditions, respectively (Fig. 2B). Optical density at 730 nm (OD_730_) increased rapidly by photomixotrophic standards increasing ~2.3 per day between 24 and 72 h when both sugars were present (Fig. 2A). Under these growth conditions, OD_730_ of 1 represents ~0.22 g of dry cell weight (gDCW) L^−1^ (ref. ^32^). This improvement is more than double the growth rate of either of the single sugar conditions, 1.2 and 0.8 d^−1^ for glucose and xylose conditions, respectively (Fig. 2A). Glucose and xylose consumption rates were ~1 g L^−1^ d^−1^ regardless of sugar conditions (Fig. 2C, D). Interestingly, the presence of one sugar did not affect the consumption rate of the other as was demonstrated to in *Synechocystis* sp PCC 6803 (ref. ^23^). The lack of CCR, the preferential consumption of one sugar in solution over another, is a desirable trait during mixed sugar fermentation^25,33^. This finding suggests that CCR is of no considerable consequence to photomixotrophic 7942 strains. The absence of sugar catabolism regulation found in heterotrophic bacterial systems such as the PTS, likely enables this strain of 7942 to catabolize mixed sugar substrates without preference while preserving the ability to fix CO_2_.

### Photomixotrophic 23BD production with high cell density

The rapid growth observed in Fig. 2A suggests that substantial carbon flux is channeled toward growth rather than product synthesis. To reduce the effects of cell growth on 23BD production, production experiments were repeated at a higher starting OD_730_ (~5), approximately equivalent to the final OD observed in the presence of both sugars after 72 h. AL3870 was cultured in 5x BG-11 at a starting OD_730_ of 5 (Fig. 2E–H). 5x BG-11 was used to provide additional nutrients, particularly nitrogen, potentially needed to support rigorous photomixotrophic growth. Sugar content was increased to 10 g L^−1^ of glucose, xylose, or both. Cultures containing mixed sugars produced 3.3 g L^−1^ of 23BD, a 3.3-fold improvement over the low OD_730_ conditions (Fig. 2F)_._ Comparatively, cultures containing only glucose made 1.8 g L^−1^, while cultures containing only xylose made 1.9 g L^−1^ (Fig. 2F). In mixed sugar conditions, xylose was consumed simultaneously with glucose (Fig. 2G, H). An extensive amount of sugar was still channeled towards biomass, as apparent from the final OD_730_ values of ~15 in the mixed sugar conditions and ~10 in the single sugar conditions (Fig. 2E). These results suggest that mixed sugar photomixotrophic growth at high culture density when supplemented with 5x BG-11 greatly improve growth and product synthesis rates. While higher cell density leads to rapid product synthesis, less light penetrance may lessen the effect of the light reactions of photosynthesis. Interestingly, yield does not appear to be dependent on light penetrance as yield is higher in high OD_730_ conditions (20% high OD_730_, 15.8% low OD_730_; Fig. 2A–H).

### Rewiring metabolism to improve photomixotrophic 23BD production from glucose and xylose

To improve photomixotrophic 23BD production from glucose and xylose, deregulation of the CBB cycle was attempted to increase the carbon flux toward RuBisCO through deletion of the Cp12 regulator and overexpression of the *prk* gene. In previous studies, these modifications drastically improved photomixotrophic 23BD production with glucose alone^22^. Cp12 is a highly conserved light dependent regulator known to bind to phosphoribulokinase (Prk), a key enzyme in the CBB cycle, and glyceraldehyde-3-phosphate dehydrogenase (GAPDH) in glycolysis^34^. The *prk* gene was placed under the IPTG-inducible *P*_LlacO1_ promoter^35^. Downstream of *P*_LlacO1_:*prk*, *galP-zwf-gnd* was placed under the IPTG-inducible *P*_trc_ promoter^36^. The DNA fragment (*P*_LlacO1_:*prk P*_trc_:*galP-zwf-gnd*) was used to delete *cp12*, creating AL4173 (Table 1). To test the effect of these genetic modifications, AL3870 and AL4173 were grown from an OD_730_ of 0.5 in 10 g L^−1^ glucose and 5 g L^−1^ xylose to mimic the lignocellulosics lysate sugar concentrations used in this study (Supplementary Table 1). AL4173 achieved 1.3 g L^−1^ of 23BD over 5 days at the theoretical maximum yield (TMY) of 37.5% while AL3870 achieved a titer of 0.7 g L^−1^ at TMY of 21.4% (Supplementary Fig. 1). Due to the production system utilizing three carbon inputs concurrently, the true yield of glucose, xylose, and CO_2_ cannot be measured. TMY is calculated based upon the yield of glucose or xylose alone. The TMY of glucose and xylose is 0.5 g 23BD per 1.0 g glucose or xylose.

Remarkably, these data suggest that deregulating the CBB cycle and introducing Prk improved the conversion of sugars to chemical products while not reducing biomass accumulation, as both strains grew equivalently (Supplementary Fig. 1A).

### Tolerance toward lignocellulosic lysate

Next, as a precursor to production experiments, the toxicity of lignocellulosic hydrolysates was tested. Lignocellulosic hydrolysate of corn stover was obtained from the National Renewable Energy Laboratory^7^. While there are many methodologies to valorize monosaccharides from lignocellulosic biomass, the lysate used in this study was processed via alkali deacetylation and mechanical milling followed by enzymatic digest^7^. The sugar content in the lysate was confirmed via high-performance liquid chromatography (HPLC) (Supplementary Table 1). *E. coli* MG1655^37^ was grown in M9 media containing 10 g L^−1^ pure glucose in the presence of lysate concentrations aligned with 0, 5, 10, 25, and 50 g L^−1^ of glucose over 24 h. While the pure glucose condition without lysate demonstrated an OD_600_ increase of ~3, the addition of any concentration of lysate limited increases in OD_600_ to less than 1 (Supplementary Fig. 2A). AL4050, a 7942 strain containing both sugar catabolism pathways but not the 23BD production pathway (Table 1), was grown in BG-11 media containing lysate concentrations with 0, 5, 10, 25, and 50 g L^−1^ of glucose under constant illumination. To account for the slower growth of AL4050 than *E. coli*, this assay was performed over 72 h. AL4050 grew best in cultures containing 5 g L^−1^ lysate glucose, with a change in OD_730_ of 0.52 (Supplementary Fig. 2B). Minor growth penalties were observed in cultures containing 10 and 25 g L^−1^ lysate glucose, with a change in OD_730_ of 0.20 and 0.27, respectively. Interestingly, cultures containing 0 g L^−1^ lysate glucose (without lysate) and 50 g L^−1^ lysate glucose were most inhibited in growth, with a change in OD_730_ of ~0.3. These results indicated that 7942 is inherently tolerant to the lignocellulosic lysates even at high concentrations, while *E. coli* is sensitive or inhibited by some molecules in the lignocellulosic lysates.

### Photomixotrophic production of 23BD utilizing lignocellulosic lysate

**Fig. 3:**
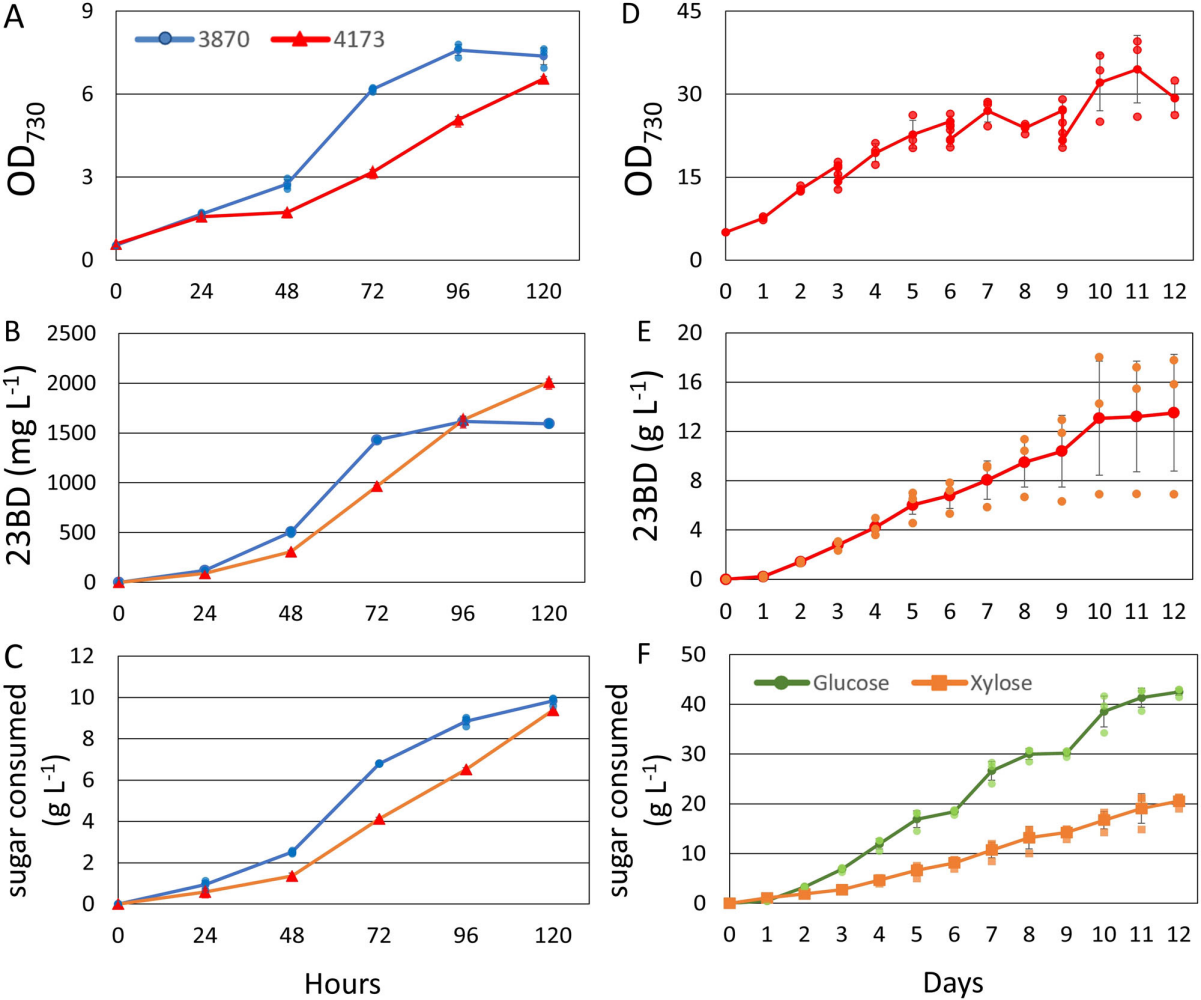
Photomixotrophic 2,3-butanediol production from lignocellulosic lysate. **A**–**C** Cells were cultured from a starting OD_730_ of 0.5 in 1x BG-11 with lignocellulosic lysate containing ~10 g L^−1^ glucose and ~5 g L^−1^ xylose. Growth (**A**), 23BD production (**B**) and total sugar consumption (**C**) of AL3870 (blue) and AL4173 (red) were monitored over 120 h. **D**–**F** AL4173 was cultured from a starting OD_730_ of 5.0 in 5x BG-11 with lysate containing ~15 g L^−1^ glucose and ~7 g L^−1^ xylose. Growth (**D**), 23BD production (**E**), and sugar consumption (**F**; glucose (green), xylose (orange)) were monitored over 12 days. *N* = 3 biological replicates; error bars represent standard deviation.

AL3870 and AL4173 were grown in media containing lysate corresponding to ~10 g L^−1^ of glucose and ~ 5 g L^−1^ xylose in the same manner as in Fig. 2. Both strains reached a higher OD_730_ over 5 days when grown in lysate as compared to just glucose and xylose. AL3870 reached an OD_730_ of 7.3 while AL4173 reached an OD_730_ of 6.6 over 5 days (Fig. 3A). 23BD production improved for both strains when grown in lysate, with AL3870 producing 1.6 g L^−1^ over 5 days at a TMY of 32.6% and AL4173 producing 2.0 g L^−1^ over 5 days at a TMY of 42.6% (Fig. 3B, C). This improvement in yield and 23BD production rate may be due to additional nutrient sources present in the lysate not found in standard BG-11. Media containing lysate-derived sugars appears to enhance growth and productivity rather than impede it, as it does in *E. coli* (Supplementary Fig. 2A), suggesting 7942 is a well-suited production host when using lysate as a feedstock.

To examine 23BD production with lysate under high cell density conditions, cells were grown from a starting OD_730_ of 5.0 in 5x BG-11 over 12 days in a tissue culture flask under constant illumination. Cultures were spun down for full media replacement every three days. A final 23BD titer of 13.5 g L^−1^ was achieved at a TMY of 42% while consuming 42 g L^−1^ of glucose and 20.5 g L^−1^ of xylose (Fig. 3D–F). A maximum OD_730_ of 35 was reached on the 11^th^ day of growth (Fig. 3D). The 23BD production rate of over 1 g L^−1^ day^−1^, is close to competing with 23BD production rates in *E. coli* which range from 1.3–6.4 g L^−1^ day^−1^ in minimum media with various sugar substrates under shake flasks conditions^38^. Constant concurrent sugar consumption and rapid growth rate for a photosynthetic microbe highlight the strength of 7942 as a photomixotrophic production host utilizing hydrolysate-based feedstocks.

While these data highlight the potential of photomixotrophic chemical production catabolizing multiple carbon sources concurrently, understanding how each of these carbon sources are utilized in downstream metabolism is essential to further improve upon this system and understand bottlenecks to inform additional metabolic engineering. Stationary and nonstationary ^13^C labeling of carbon feedstocks are well-established methods to determine metabolite flux and accumulation of a carbon source in cyanobacteria and has been demonstrated to inform metabolic engineering efforts in cyanobacteria^39–41^. This photomixotrophic production system is uniquely difficult to probe with ^13^C labeling techniques as multiple carbon sources must be traced in parallel. Parallel labeling experiments have precedent but are experimentally and computationally resource intensive^42^. This production system provides an opportunity to improve parallel ^13^C methodologies as it is not limited by CCR as most common production hosts are and can additionally fix CO_2_. Further work on the photomixotrophic system described in this work is an opportunity to understand the metabolic considerations of concurrent sugar utilization, provides a proving ground for multisugar parallel ^13^C labeling technology, and would further inform future efforts to efficiently utilize lignocellulosic lysates.

## Conclusion

In this study, 7942 was engineered to photomixotrophcally grow and produce 23BD from a mixed-sugar substrate derived from hydrolyzed waste biomass. Our strain managed to consume glucose and xylose at similar rates without preference, the lack of carbon catabolite repression is extremely important for the utilization of hydrolyzed waste biomass. The tolerance of a photomixotrophic host in extremely high lysate concentrations were demonstrated even when traditional fermentation hosts such as *E. coli* could not survive. 7942 was found to grow better in the presence of lysate than without and could consume multiple sugars concurrently with no apparent penalty to growth or production rate. Further modifications of the CBB led to a 16.1% increase in yield and increased final titer by 85.7% in pure sugar conditions. Rapid growth and 23BD product synthesis over 1 g L^−1^day^−1^ was observed at an OD_730_ of over 30 during photomixotrophic culturing in the presence of lysate. Previous work has shown that TMYs of over 100% are possible in photomixotrophic production systems, indicating that further optimization of growth conditions could greatly improve yield over those reported here^22^. While this study provides a foundation for further elucidation of lignocellulosic photomixotrophy, future studies utilizing omics analyses such as ^13^C carbon labeling are critical to further understand and improve upon this system. The advent of tools such as CRISPR/cas9-like gene editing in cyanobacterium and algae suggest a bright future is possible for photomixotrophic production hosts^43^. Despite these recent advances, photosynthetic bioreactor design and industrial scale photosynthetic microbe cultivation remains a major challenge. This work highlights the potential of 7942 to be further utilized and improved upon as a platform organism for products synthesized from lignocellulosic lysate.

## Methods

### Reagents

The following reagents were obtained from Sigma-Aldrich: glucose, xylose, cycloheximide, 23BD, isopropylthio-β-galactoside (IPTG), gentamycin, and spectinomycin. Kanamycin was obtained from Fischer Scientific. Phusion polymerase was purchased from New England Biolabs. HiFi ToughMix polymerase was purchased from QuantBio. All oligonucleotide synthesis was done by Integrated DNA technologies. All sequencing was performed by Genewiz Azenta.

### Plasmid construction

All plasmids and primers used in this study are listed in Tables 2 and 3, respectively. The target genes and vector fragments used to construct plasmids were amplified using PCR utilizing Phusion or HiFitoughmix polymerase. The *lacI*^*q*^; *P*_trc_: *galP-zwf-gnd* fragment for pAL1515 (addgeneID:209176) was amplified from pAL1450^22^ using the primers MK373 and MK376. The NSII Kan^R^ fragment of pAL1515 was amplified from pAL1200 using MK375 and MK374. The *P*_trc_: *galP-zwf-gnd* fragment for pAL2301 (addgeneID:209177) was amplified from pAL1450 using TT628 and TT629. The *cp12*:: *P*_LlacO1_: *prk* Kan^R^ fragment was amplified from pAL2141 (unpublished) using TT632 and TT633. The resulting fragments were assembled by sequence and ligation-independent cloning^44^.

**Table 2:** Plasmids used in this study.

Plasmid	Description	Source
pAL70	NSI:: *lacI*^*q*^; *P*_trc_: *xylE-xylA-xylB*; Spec^R^	McEwen 2013^18^
pAL1136	NSIII:: *lacI*^*q*^; *P*_trc_: *alsD-alsS-adh;* Gent^R^	Oliver 2013^26^
pAL1200	NSII:: *lacI*^*q*^; *P*_trc_: *galP* Kan^R^	McEwen 2013^18^
pAL1450	NSI:: *lacI*^*q*^; *P*_trc_: *galP-zwf-gnd*; Spec^R^	Kanno 2017^22^
pAL1515	NSII:: *lacI*^*q*^; *P*_trc_: *galP-zwf-gnd*; Kan^R^	This study
pAL2141	*cp12*:: *P*_LlacO1_: *prk*; Kan^R^	Kanno 2017^22^
pAL2301	*cp12*:: *P*_LlacO1_: *prk P*_*trc*_:*galP-zwf-gnd*; Kan^R^	This study

**Table 3:** Oligonucleotides used in this study.

Name	Sequence 5′ to 3′	Plasmids produced
TT628	gtttgtcggtgaacgctctccgactgcacggtgcaccaatg	pAL2301
TT629	gtgagcgttgattgaggtgagcctctagaacgcgtgagagcg	pAL2301
TT632	ctctcacgcgttctagaggctcacctcaatcaacgctcacc	pAL2301
TT633	attggtgcaccgtgcagtcggagagcgttcaccgacaaacaac	pAL2301
MK373	ttgatgcctctagaacgcgtgagagcgttcaccgacaaac	pAL1515
MK374	gtttgtcggtgaacgctctcacgcgttctagaggcatcaa	pAL1515
MK375	gactggaaagcgggcagtgaattaatgcagcttaaggttg	pAL1515
MK376	caaccttaagctgcattaattcactgcccgctttccagtc	pAL1515

### Strain construction

The strains used in this study are listed in Table 1. Transformation and integration via double homologous recombination were performed as previously described^45^. In brief, cells at OD_730_ ∼0.4 were collected from 2 ml of culture by centrifugation, washed, and concentrated in 300 μl of BG-11 medium. After adding plasmid DNA (2 μg) to the concentrated cells, the tube was wrapped in foil and incubated overnight at 30 °C. Cells were plated on BG-11-agar solid media containing appropriate antibiotics and incubated at 30 °C under constant light until colonies appeared. Complete chromosomal segregation of the introduced fragments was achieved through propagation of multiple generations on selective agar plates. Correct segregated double recombinants were confirmed by PCR and Sanger sequencing of these PCR products.

### Culture conditions

Unless otherwise specified, *S. elongatus* cells were cultured in BG-11 medium^46^ with the addition of 50 mM NaHCO_3_. Cells were grown at 30 °C with rotary shaking (100 rpm) and light (30 mmol photons µm^−2^ 2 s^−1^ in the PAR range) provided by 86 cm 20 W fluorescent tubes. Light intensity was measured using a PAR quantum flux meter (Model MQ-200, Apogee Instruments). Cell growth was monitored by measuring OD_730_ in a Microtek Synergy H1 plate reader (BioTek). Antibiotics concentrations were as follows: cycloheximide (50 mg L^−1^), spectinomycin (20 mg L^−1^), kanamycin (20 mg L^−1^), gentamycin (10 mg L^−1^). Prior to 23BD production, colonies were inoculated in BG-11 medium containing 50 mM NaHCO_3_ and appropriate antibiotics and grown photoautotrophically. These cultures were then centrifuged at an RCF of 4000 x g and resuspended in fresh media to achieve either OD_730_ 0.5 (10 mL culture in a 20 mL glass culture tube) or 5 (50 mL culture in a 250 mL baffled flask or a 250 mL tissue culture flask). Every 24 h, 1 mL of the culture volume was removed, the pH was adjusted to 7.0 with 3.6 N HCl (~40 µL for 10 mL cultures and ~240 µL for 50 mL cultures) and sample volume was replaced with pH 7 production media containing appropriate bicarbonate to reach a concentration of 20 mM (1 mL of 200 mM bicarbonate replacement media to replace 1 mL of sample from a 10 mL culture). For high cell density experiments, cells at the exponential growth phase were adjusted to an OD_730_ of 5.0 in 5 x BG-11 media, which was composed of quintupled 1x BG-11 medium component concentrations except for HEPES-KOH and A5 trace metals, which remained unchanged, and 20 mM NaHCO_3_, 1 mM IPTG, 10 mg L^−1^ thiamine and appropriate antibiotics including cycloheximide in a 250 mL tissue culture flask. Once every three days the culture was spun down, the old media supernatant was removed, and the pellet was resuspended with 50 mL of fresh media.

### Quantification of glucose and xylose

Glucose and xylose in culture supernatant were quantified using a HPLC (LC-20AB, Shimadzu) equipped with a Fast Acid Analysis Column (Biorad, Hercules) and a differential refractive detector (RID-10A). 5 mM H_2_SO_4_ served as the mobile phase at a flow rate of 0.6 mL min^−1^ at 65 °C for 15 min.

### 23BD quantification

Culture supernatant samples were analyzed by a gas chromatograph (GC-2010, Shimadzu) equipped with a flame ionization detector and DB-WAX column (30 m, 0.32 mm internal diameter; Agilent Technologies). The GC oven temperature was held at 105 °C for 1 min, increased with a gradient of 20 °C/min until 225 °C and held for 3 min. The temperature of the injector and detector was 250 °C.

### Lignocellulosic lysate production-

Lignocellulosic lysate was generated in accordance with the process described by Chen et al.^7^, with the following modifications: Deacetylation was conducted at 90 °C, no secondary milling (Szego) was performed, total enzyme loading was 25 mg g^−1^, the enzymatic hydrolysate was clarified in a pilot-scale cross flow filter (PALL) using a nominal 0.1 µm pore size sintered metal membrane, the clarified enzymatic hydrolysate (permeate) was concentrated under vacuum ( ~ 22” Hg of vacuum) in a forced circulation evaporator (Ender Process Equipment Corp.) at ~60‒65 °C with 1.0‒1.5 h of retention.

### Statistics and reproducibility

No statistical method was used to determine the necessary sample size for each experiment. An *n* = *3* of biological replicates was used for all experiments. A replicate represents sequence confirmed biological replicates of *Synechococcus elongatus* PCC 7942. Data analysis was done in Microsoft excel. Data is expressed as mean ± standard deviation (SD).

### Reporting summary

Further information on research design is available in the Nature Portfolio Reporting Summary linked to this article.

### Supplementary information

Supplementary Informaton

Description of Additional Supplementary Files

Supplementary Data 1

Reporting Summary

## Data Availability

The datasets generated in this study are available from the corresponding author on reasonable request. Source data are provided with this paper (Supplementary Data 1). pAL1515 and pAL2301 can be found with the identifiers (addgeneID:209176) and (addgeneID:209177).
