# Supplementary material for: Utilization of lignocellulosic hydrolysates for photomixotrophic chemical production in Synechococcus elongatus PCC 7942
Source: Commun Biol. 2023 Oct 9;6:1022. doi: 10.1038/s42003-023-05394-w (PMC10562401; doi:10.1038/s42003-023-05394-w)
Supplement: Supplementary file 1 — Supplementary Informaton [file 42003_2023_5394_MOESM1_ESM.pdf]

Supplementary Information

Utilization of lignocellulosic hydrolysates for photomixotrophic chemical production in *Synechococcus elongatus* PCC 7942

Jake N. Gonzales, Tanner R. Treece, Stephen P. Mayfield, Ryan Simkovsky, and Shota Atsumi

Table of Contents

Supplementary Table 1. Lignocellulosic lysate contents

Supplementary Figure 1. Photomixotrophic 2,3-butanediol production from mixed sugars.

Supplementary Figure 2. Lignocellulosic lysate tolerance in *E. coli* and 7942

Supplementary Table 1. Lignocellulosic lysate contents

Cellobiose	Glucose	Xylose	Arabinose	Lactate	Acetate	HMF	Furfural
ND	360	187	16	15	ND	ND	ND

(g L^{-1}); ND, not detectable

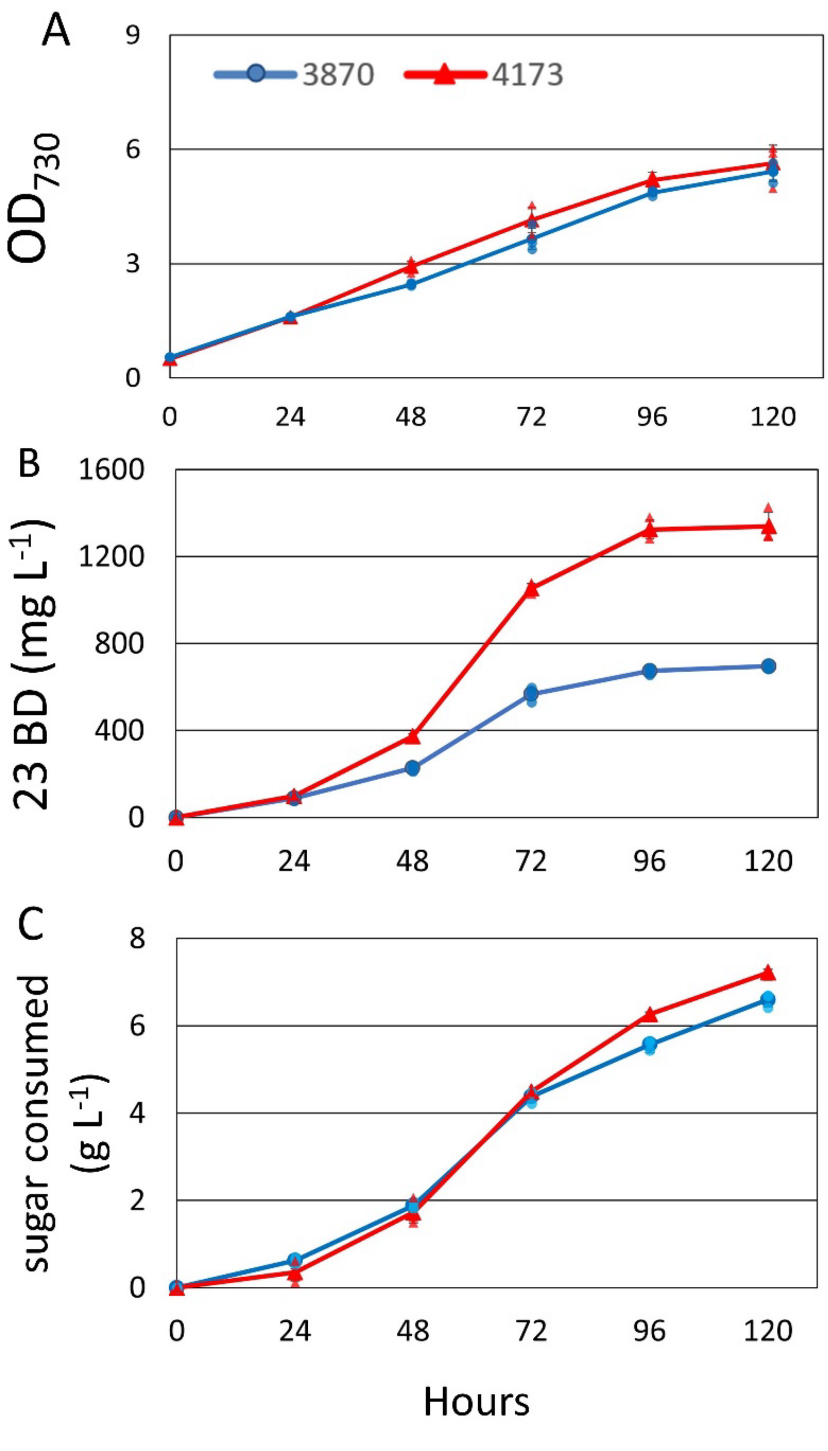

Supplementary Figure 1. Photomixotrophic 2,3-butanediol production from mixed sugars.

Cells were cultured from a starting OD_{730} of 0.5 in 10 mL of 1 x BG-11 mixed with 10 g L^{-1} glucose and 5 g L^{-1} xylose. Growth (**A**), 23BD production (**B**) and total sugar consumption (**C**) of AL3870 (blue) and AL4173 (red) were monitored over the course of 120 h. *N*=3 biological replicates; error bars represent standard deviation.

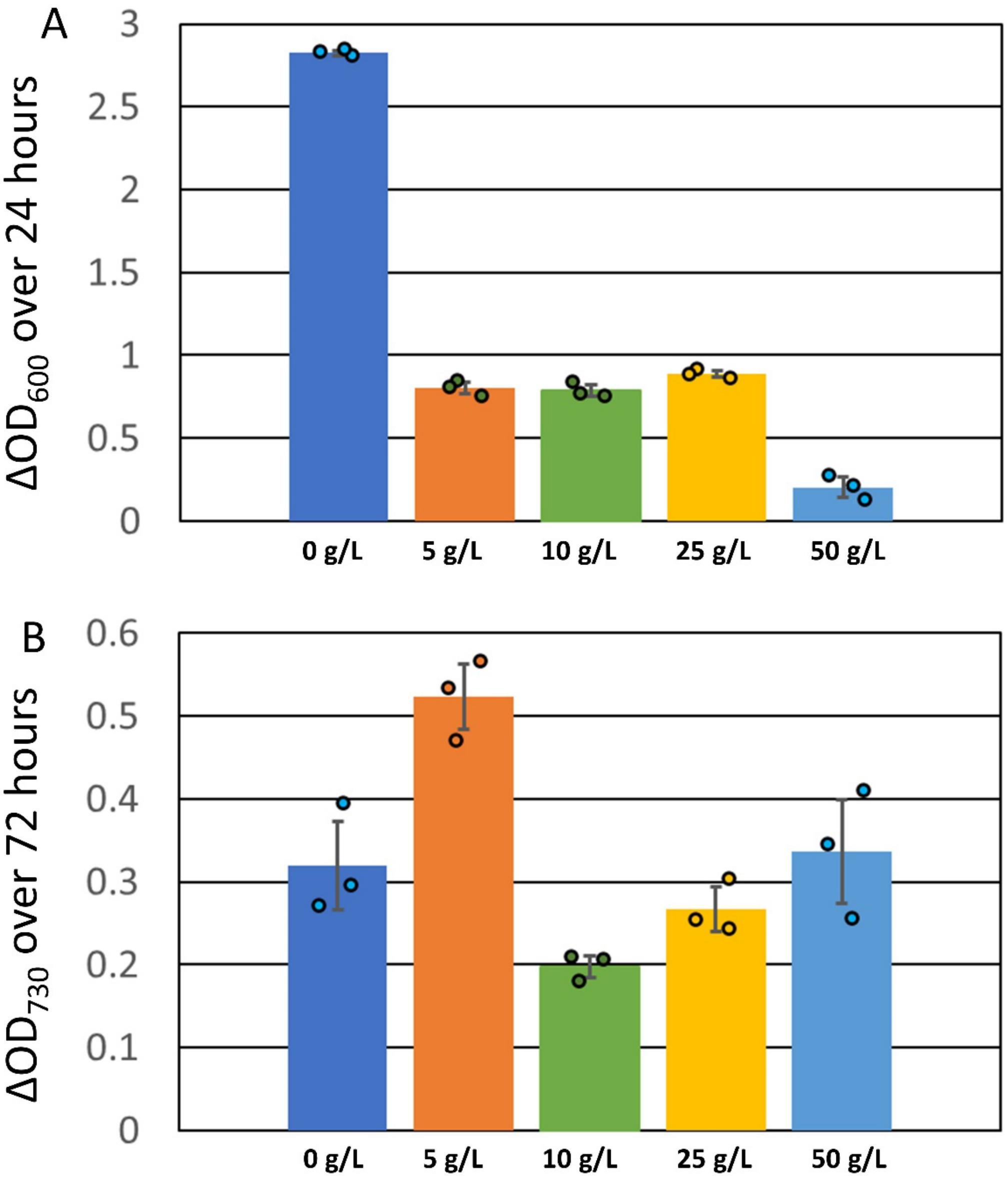

Supplementary Figure 2. Lignocellulosic lysate tolerance in *E. coli* and 7942.
E. coli strain MG1655 and AL4050 (Table 1) were grown in M9 or BG-11 media in the presence of lignocellulosic lysate corresponding to glucose concentrations of 0 (blue), 5 (red), 10 (green), 25 (purple), or 50 (cyan) g L^{-1}. Change in OD_{600} or OD_{730} was observed over the span of 24 hours or 72 hours respectively. *N*=3 biological replicates; error bars represent standard deviation.
