# Supplementary material for: Utilization of lignocellulosic hydrolysates for photomixotrophic chemical production in Synechococcus elongatus PCC 7942
Source: Commun Biol. 2023 Oct 9;6:1022. doi: 10.1038/s42003-023-05394-w (PMC10562401; doi:10.1038/s42003-023-05394-w)
Supplement: Supplementary file 2 — Description of Additional Supplementary Files [file 42003_2023_5394_MOESM2_ESM.pdf]

Description of Additional Supplementary Files

File name: Supplementary Data 1
Description: Source data for all figures
